# Proto-oncogene tyrosine-protein kinase SRC (Src) inhibition in microglia relieves neuroinflammation in neuropathic pain mouse models

**DOI:** 10.1080/21655979.2021.2008694

**Published:** 2021-12-01

**Authors:** Yuanxing Cai, Jing Xu, Qinghao Cheng

**Affiliations:** Department of Anesthesiology, Emergency General Hospital, Beijing, China

**Keywords:** Neuropathic pain, Src, PP2, microglia, neuroinflammation

## Abstract

Chronic neuroinflammation is an important factor in the development of neuropathic pain (NP). Excess microglia activation releases a mass of pro-inflammatory cytokines during neuroinflammation process, leading to a constant painful irritation of the sensory nerve. Src belongs to a non-receptor tyrosine kinase associated with sarcoma, whereas the role of Src in neuropathic pain is controversial. We designed to testify the inflammation-regulatory role of Src in the lipopolysaccharide (LPS)-induced BV2 microglia line and the mouse model of neuropathic pain by partial sciatic nerve ligation (PNL). In BV2 microglia, Src expression was inhibited using a Src family kinase inhibitor PP2 after LPS induced inflammatory response. *In vivo*, the neuropathic pain in mice was induced by PNL surgery and then treated with PP2. The neuroinflammation level *in vitro* was detected by enzyme-linked immunosorbent assay (ELISA), immunofluorescence (IF), trans-well and Western blotting (WB) assays, *in vivo* was examined in PNL mice using immunohistochemistry (IHC) and IF. Finally, mechanical allodynia and thermal hyperalgesia assays were used to access the functional evaluation. Inhibition of Src was decreased microglial inflammation and migration after LPS stimuli. Mechanistically, the expression of nuclear factor kappa B (NF-κB) pathway decreased after Src inhibition. The data *in vivo* showed that the decrease expression of Src reduced neuroinflammation and the amount of microglia in spinal dorsal horn (SDH), the mechanical allodynia of mice thereby attenuated after Src inhibition. These results indicated that the inhibition of Src took a protective effect in neuropathic pain mouse models *via* reducing microglia-induced neuroinflammation.

## Introduction

Neuropathic pain (NP), a neuroinflammation-related progressive neurological disorder, is characterized by ambulatory pain accompanied with paresthesia and dysesthesia [1]. The sensitization of central and peripheral neurons caused by the chronic inflammation in the spinal dorsal horn (SDH) maintains the process of NP [2]. Thereinto, microglia serving as a promoter in the SDH may involve in the pathogenesis of NP [3]; however, the explicit mechanism is still not synthetically understood. Identifying new targets leading to NP is a crucial goal of drug development. Following peripheral nerve injury, injured sensory neurons release cytokines including colony-stimulating factor-1, which trigger microglia accumulation and activation in the SDH [4,5]. The activated microglia with reactive astrocytes then produce various extracellular factors to exacerbate NP *via* diverse paracrine pathways [6–8]. Excess pro-inflammatory mediators, such as tumor necrosis factor (TNF)α, interleukin-1β (IL-1β) secreted by activated microglia contribute to the development of NP [9,10]. Preclinical and clinical trials thereupon were conducted to examine the effectiveness of anti-inflammatory reagents in treatment of NP.

Proto-oncogene tyrosine-protein kinase SRC (Src) is deemed as a non-receptor tyrosine kinase that is widely expressed in various cells [11,12]. Notably, Src is a primary regulator of numerous signaling pathways regulating and maintaining biological activities, such as cell metabolism, cellular contact, and cell migration; importantly controlling signal transduction associated with inflammatory responses [13–15]. Previous studies [16,17] have reported that activated Src was at high level in various tumor tissues. Importantly, also some researchers proved that the Src-related signaling participated in the factors-regulated interaction between inflammatory cells and tumor cells [18,19]. Therefore, increasing scientists start to investigate whether Src is related to some inflammatory diseases elsewhere. Socodato and his workmates suggested that the expression of Src is sufficient for the development of microgliosis [20]. A recent study showed that Src inhibition exerted anti-inflammatory effects in Aβ stimulated microglia [21]. Together with the above, we thereby hypothesized that a proper modulation of Src tyrosine kinase expression might control microglial inflammation, which potentially ameliorates the level of neuroinflammation and be beneficial to the remission of NP.

In our study, we hypothesized that Src plays a positive role in inflammation response during NP. To investigate whether pharmacological inhibition of Src attenuates microglia-induced inflammation during NP, we utilized the lipopolysaccharide (LPS)-stimulated BV2 cell and partial sciatic nerve ligation (PNL)-treated mouse to testify the role of Src inhibition and the underlying mechanisms in neuroinflammation both *in vitro* and *in vivo*.

## Materials and methods

### Cell treatment

A BV2 cell line was obtained from the Cell Culture Center of Chinese Academy of Medical Sciences (Shanghai, China). The cells were cultured using dulbecco’s modified eagle medium (DMEM, Servicebio, Wuhan, China) containing 10% fetal bovine serum (FBS, Gbico, Rockville, MD, USA), 100 IU/mL penicillin, and 100 μg/mL streptomycin (Invitrogen, Carlsbad, CA, USA) at 37°C in an atmosphere of 5% CO_2_. Src kinase inhibitor PP2 (MCE, Monmouth Junction, NJ, USA) dissolved in dimethyl sulfoxide (DMSO, Solarbio, Beijing, China) was prepared for a concentration of 200 mM and diluted with DMEM to decrease DMSO concentration lower than 0.1%. PP2 administration at specific concentration was treated BV2 cell for 24 h. Then the treated cells were activated inflammatory response using lipopolysaccharide (LPS, 100 ng/mL, Sigma, St. Louis, MO, USA) for 24 h when the confluency reached to 90%.

### Cell viability assay

After PP2 treatment at concentration range of 5–200 µM for 24 h, the cells were harvested and detected cell viability using a Cell counting kit-8 Kit (Dojindo, Kumamoto, Japan) in accordance with the manufacturer’s protocol.

### Experimental animals of PNL treatment

Total of 24 male C57BL/6 J mice (8–12 weeks, 22 g) were purchased from Lianyungang First People’s Hospital Animal Center. The animals were housed in adaptive environment (22–26°C, 55–65% humidity, and 12 h/12 h light/dark cycle) and provided with available food and tap water. This study was approved by the Animal Ethics Committee of Lianyungang First People’s Hospital Animal Center in accordance with the Guide for the Care and Use of Laboratory Animals. They were randomly divided into the Sham group, PNL group, and PNL+PP2 group. The mice were anesthetized with inhalational isoflurane by 5% for induction and 2% for maintenance, and then the mouse's right thigh was dissected to expose the sciatic nerve. The right sciatic nerve including the dorsal one-third to one-half, approximately, was ligated by a 9–0 nylon suture followed by disinfection and suture of wound [22]. The sham group was performed the same operations except for the PNL. Mice were first treated with PP2 (i.p.) 6 h after PNL operation. From day 2 to day 21, mice were continuously treated with PP2. At days 2, 3, 7, 10, 14, and 17, 21, we detected the behavior using paw withdrawal threshold test (Figure 3(a)).

### Trans-well assay

Cells were suspended in FBS-free DMEM and added into the upper chamber with a volume of 200 µL. The DMEM with FBS was added in the under layer. After 24 h, we harvested the cells using 4% paraformaldehyde and dyed the cells using crystal violet for 15 min. Then the cells on the upper chamber were wiped by a cotton swab and the cells on lower chamber were captured with a microscope (OLYMPUS, Tokyo, Japan).

### Reverse transcription-quantitative polymerase chain reaction (RT-qPCR)

The Cell and tissue were added into a TRIzol reagent (Invitrogen, Carlsbad, CA, USA). cDNA synthesis was carried out using a PrimeScript RT Reagent Kit (TaKaRa, Shiga, Japan). Measurement of RNAs was performed using a chimeric dye SYBR® Premix Ex Taq ^TM^ II Kit (TaKaRa, Shiga, Japan) at a Thermal Cycler Dice Real-Time System (TP800, TaKaRa, Shiga, Japan). Glyceraldehyde 3-phosphate dehydrogenase (GAPDH) was used to normalize the RNAs level according to the 2-^ΔΔCt^ method [23]. Primers are shown as follows: GAPDH, Forward Primer: TGACCTCAACTACATGGTCTACA, Reverse Primer: CTTCCCATTCTCGGCCTTG; iNOS, Forward Primer: GTTCTCAGCCCAACAATACAAGA; Reverse Primer: GTGGACGGGTCGATGTCAC; COX-2, Forward Primer: TTCCAATCCATGTCAAAACCGT, Reverse Primer: AGTCCGGGTACAGTCACACTT; TNF-α, Forward Primer: CTGAACTTCGGGGTGATCGG, Reverse Primer: GGCTTGTCACTCGAATTTTGAGA; IL-6, Forward Primer: TCTATACCACTTCACAAGTCGGA, Reverse Primer: GAATTGCCATTGCACAACTCTTT.

### Enzyme-linked immunosorbent assay (ELISA)

The cells and tissue were collected and added into radioimmunoprecipitation assay (RIPA) lysis (Solarbio, Beijing, China) were then centrifuged for 10 min to gather the supernatant. Then the samples were used to verify protein concentration using bicinchoninic acid (BCA) method [24] (YiFeiXue, Shanghai, China). Further steps were performed using ELISA Kits (YiFeiXue, Shanghai, China) in accordance with the manufacturers’ protocols. The OD value was measured at the wavelength.

### Immunofluorescence (IF) and immunohistochemical (IHC) staining

Paraffin sections of spinal cord tissue were conducted antigen blocking using 3% BSA blocking buffer for 1 h at room temperature. The sections were incubated with primary rabbit antibodies to phosphorylated Src (p-Src, 1:100, Cell Signaling Technology, Danvers, MA, USA), microglia marker ionized calcium-binding adaptor molecule-1 (IBA-1, 1:100, Proteintech, Rosemont, IL, USA), inducible nitric oxide synthase (iNOS) (1:100, Proteintech, Rosemont, IL, USA), and cyclooxygenase 2 (COX-2, 1:200, Abcam) at 4°C overnight. Sections were washed three times, then were performed using fluorescence secondary antibodies for 1 h in dark at room temperature or diaminobenzidine (DAB) treatment for coloration. Images were collected using a microscope system (OLYMPUS, Tokyo, Japan).

### Western blotting (WB) assay

BV2 microglia or spinal cord tissues were collected in RIPA lysis supplemented inhibitors of phosphatase and protease. Following measurement of protein concentrations using a BCA Kit, equal sample loading was used to sodium dodecyl sulfate polyacrylamide gel electrophoresis. Then, polyvinylidene fluoride membrane (Millipore, Billerica, MA, USA) was utilized to transfer protein. Then the membranes were incubated with the following antibodies: GAPDH (1:10,000, Proteintech, Rosemont, IL, USA), Src (1:1000, Cell Signaling Technology, Danvers, MA, USA), p-Src (1:1000, Cell Signaling Technology, Danvers, MA, USA), NF-κB (1:1000, Cell Signaling Technology, Danvers, MA, USA), and histone H3 (1:1000, Cell Signaling Technology, Danvers, MA, USA) 4°C overnight; then these were probed with HRP goat anti-rabbit IgG (1,10,000, Proteintech, Rosemont, IL, USA) at room temperature for 1 h. GAPDH was used for normalization. The blots were captured using an enhanced chemiluminescence system and analyzed using the ImageJ software (NIH, Bethesda, MD, USA).

### Behavioral testing

All mice were tested on days 2, 3, 7, 10, 14, 17, and 21 post PNL or sham operation. Paw withdrawal threshold (PWT) was assessed to measure mice in response to the mechanical stimuli by pain gauge measurement (von Frey, Woodland Hills, CA, USA). The mice were placed individually in transparent plastic cages with wire mesh floor and kept for 30 min. The stimuli were transferred to the plantar surface of the hind paw with the calibrated Electronic von Frey filament using the up-down method.

### Statistical analysis

We utilized Statistical Product and Service Solutions (SPSS) 21.0 software (Chicago, IL, USA) to do statistical analysis. Differences between two groups were analyzed by using the Student’s t-test. Comparison between multiple groups was done using One-way ANOVA test followed by Post Hoc Test (Least Significant Difference). Repeated-measures ANOVA followed by Bonferroni post hoc test was used to analyze at different time points. *p* < 0.05 was considered as significant difference.

## Results

Herein, we hypothesized that inhibition of Src by PP2 could ameliorate inflammation and NP degree. To trace the role of Src inhibition in NP, we mimicked inflammation model using LPS-induced microglia in vitro and established NP model by PNL surgery in vivo. We further detected the effect and molecular mechanism of Src inhibitor PP2 in inflammatory microglia and in NP mice.

### Src inhibitor reduces BV2 microglial activation by LPS stimulation

**Figure 1.**
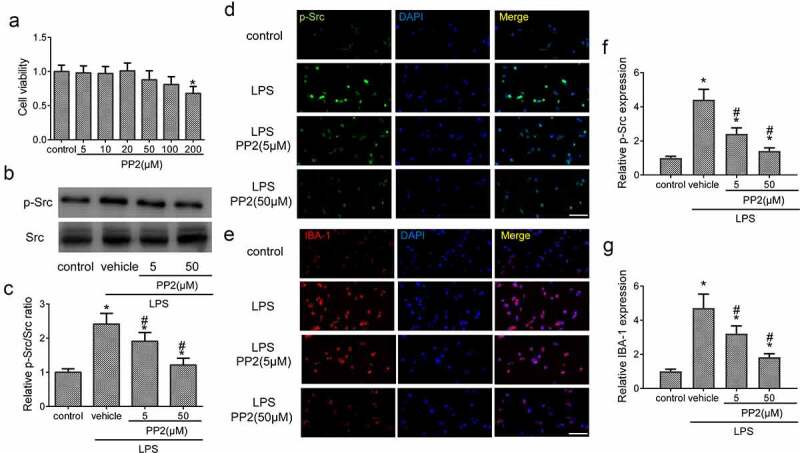
Src inhibitor reduces LPS-induced the activation of BV2 microglia. (a) Cell viability of BV2 microglia after 5, 10, 20, 50, 100 and 200 µM PP2 treatment for 24 h. (b) The Western blotting of p-Src and Src in LPS-stimulated microglia after 5 or 50 µM PP2 treatment for 24 h. (c) The analysis of the p-Src/Src ratio. (d) The IF images of p-Src in control, LPS, LPS+PP2 (5 µM) and LPS+PP2 (50 µM) group; amplification: 400 × . (e) The IF images of IBA-1 in control, LPS, LPS+PP2 (5 µM) and LPS+PP2 (50 µM) group; amplification: 400×; Scale bar = 100 μm. ‘*’ means *p* < 0.05 vs. the control group, ‘#’ means *p* < 0.05 vs. the LPS+vehicle group

First, the cell viability experiment was conducted to assess the toxic effect of PP2 treatment at the concentration range from 5 µM to 200 µM for 24 h. The results showed that the concentration of PP2 from 5 to 50 µM hardly exert a toxicity on microglial cell viability with 100 ng/mL LPS treatment or not; however, a significant cytotoxicity of PP2 was exhibited in 200 µM (Figure 1(a)). Hence, we selected the concentration at 5 and 50 µM for PP2 treatment in our study. Furthermore, the level of Src phosphorylation in microglia after PP2 administration was measured. WB assay displayed that PP2 treatment reduced the expression of p-Src (Figure 1(b,c)) after LPS treatment in microglia. Consistently, IF staining showed that the inhibitor of Src significantly decreased the p-Src fluorescence intensity in LPS treated BV2 microglia (Figure 1(d,f)). Then the expression of IBA-1 in microglia was examined by IF, showing that PP2 treatment markedly inhibited the expression of IBA-1 after LPS utilization (Figure 1(e,g)). These results verify that PP2 as a potent Src inhibitor attenuates LPS-induced activation of microglia.

### Src inhibition inhibits microglia-driven inflammation via blocking NF-κB activity

**Figure 2.**
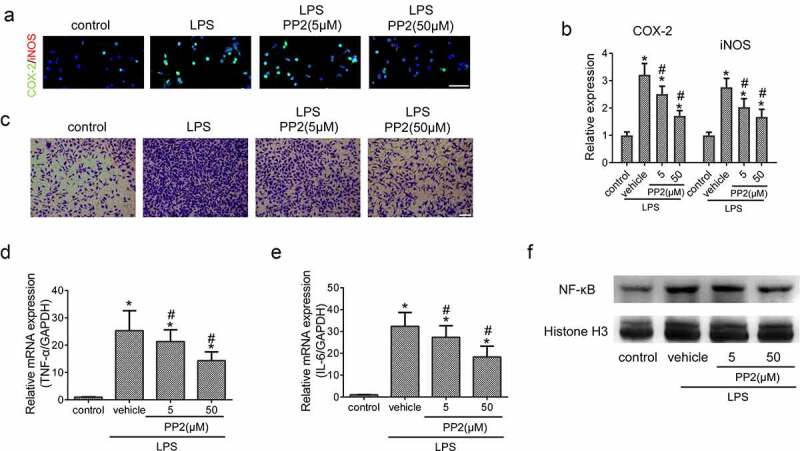
Src inhibition inhibits microglia-driven inflammation *via* blocking NF-κB activity. (a) The double IF staining including COX-2 and iNOS in LPS-stimulated microglia after 5 or 50 µM PP2 treatment for 24 h, amplification: 400×; Scale bar = 100 μm. (b) The representative images of microglial trans-well assay after LPS stimuli with PP2 treatment or not; Scale bar = 100 μm. The mRNA levels of TNF-α (c) and IL-6 (d) in LPS-stimulated microglia after 5 or 50 µM PP2 treatment for 12 h. (e) The Western blotting assay of NF-κB in microglial nucleus after LPS stimuli with PP2 treatment or not. ‘*’ means *p* < 0.05 vs. the control group, ‘#’ means *p* < 0.05 vs. the LPS+vehicle group

Next, to certain the effective role of Src inhibition in microglia-driven inflammation, the expression of COX-2 and iNOS in LPS treated microglia was detected using IF after PP2 administration. The images showed that LPS treatment led to an increase of the COX-2 and iNOS expression in microglia, but the increased levels were reduced under PP2 administration (Figure 2(a,b)). Furthermore, the number of migrant microglia treated with PP2 were decreased significantly after 24 h LPS induction (Figure 2(c)). The mRNA levels including TNF-α and IL-6 were also reduced in LPS-induced microglia subjected to PP2 (Figure 2(d,e)). NF-κB pathway is a critical signaling pathway for proinflammatory process in microglia, and we thereby examined the level of NF-κB pathway following Src inhibition by PP2. WB analysis revealed that PP2 treatment reduced the nuclear translocation of NF-κB in activated microglia (Figure 2(f)). Thus, the data demonstrate that Src inhibition attenuates inflammatory response *via* inhibiting NF-κB pathway in LPS treated microglia.

**Figure 3.**
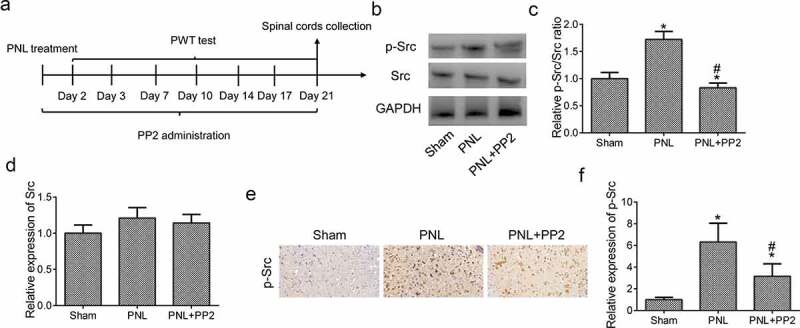
Confirmation of the efficiency of Src inhibitor PP2 in PNL-treated mice. (a) The program flowchart of NP model experiments *in vivo*. (b) The protein expression of p-Src and Src in the spinal cords of PNL-treated mice at 21 days post injury. (c) The analysis of the p-Src/Src ratio. (d) The quantitation of Src protein. (e) The IHC staining of p-Src in SDH of PNL-treated mice at 21 days post injury; amplification: 400×; Scale bar = 100 μm. (f) The quantitation of Src positive area. ‘*’ means *p* < 0.05 vs. the sham group, ‘#’ means *p* < 0.05 vs. the PNL group

### Determination of the function of Src inhibitor in PNL-treated mouse models

As shown in Figure 3(a), PNL mice were administrated with 2.5 mg/kg PP2 for 3 weeks after surgery. To confirm the function of Src inhibition by PP2 in PNL mice, the level of p-Src expression in L3-L4 spinal cords was detected using WB assay at 21 days post PNL. It was shown that the level of p-Src expression increased markedly in the PNL mice while PP2 treatment significantly reduced p-Src expression in spinal cords. There is no difference of Src expression in PNL treated mice (Figure 3(b-d)). The IHC staining was shown to be a consistent expression of p-Src with WB analysis (Figure 3(e,f)). To sum up, the above results suggest that PP2 inhibits Src phosphorylation in PNL-treated mice.

### Src inhibition reduces neuroinflammation and improves allodynia after PNL

**Figure 4.**
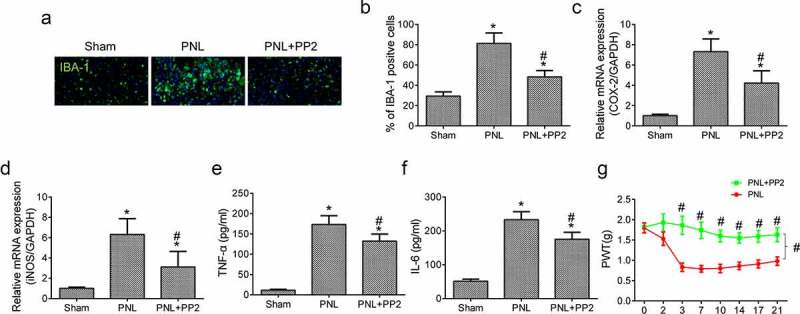
Src inhibition reduces neuroinflammation and improves allodynia after PNL. (a) The IF staining of IBA-1 in SDH of PNL-treated mice at 21 days post injury; amplification: 400×; Scale bar = 100 μm. (b) The quantitation of IBA-1 positive cells. The mRNA levels of COX-2 (c) and iNOS (d) in the spinal cords of PNL mice at 21 days post injury. The ELISA assay of TNF-α (e) and IL-6 (f) in the spinal cords of PNL mice at 21 days post injury. (g) The PWT assay of mice at 2, 3, 7, 10, 14, 17, 21 days post PNL. ‘*’ means *p* < 0.05 vs. the sham group, ‘#’ means *p* < 0.05 vs. the PNL group

Last, to determine whether Src inhibition by PP2 reduced microglia-induced neuroinflammation in spinal cords of PNL treated mice, IF assay of IBA-1 expression was performed in the cords tissue. The amount of IBA-1 positive glial cells in the SDH exhibited a prominent increase in the PNL treated mice, which was reduced remarkably by PP2 administration (Figure 4(a,b)). Moreover, the PCR result showed that the mRNA expression of COX-2 and iNOS was increased highly in tissue after PNL treatment while Src inhibition decreased the levels markedly (Figure 4(c,d)). Furthermore, the levels of TNF-α and IL-6 in PP2 treated PNL mice were prominently reduced lower than those without treatment (Figure 4(e,f)). The PP2 treated mice showed a better PWTs than the untreated mice (Figure 4(e-g)), indicating that the sensitivity to punctate stimulation of the mice was improved after PP2 administration. Collectively, the results demonstrate that Src inhibition prevents functional impairment in PNL-induced NP mice by reducing microglial neuroinflammation.

## Discussion

In the present research, our data confirmed a negative role of Src inhibition in microglia activation and elaborated the effect of Src on microglial induced neuroinflammation. We proved that PP2-induced Src inhibition not only alleviated the degree of inflammatory mediators but also restrained the infiltrating ability of microglia. Importantly, inhibition of Src could significantly correct the disorder of sensitivity in lower limbs of PNL treated mice. Hence, our results testified that Src might become a novel target in investigation of NP treatment. Besides, other researchers have suggested that Src plays an important role in NP pathogenesis. Felice et al. showed that Inhibition of non-receptor tyrosine kinase Src controls N-methyl-D-aspartate receptor activity and reduces the hypersensitivity associated with neuropathic and inflammatory pains [25]. Likewise, Chen and Marvizón reported that a Src family kinase plays a role in a model of inflammatory and NP [26]. Chronic neuroinflammation, owing to vast microglial accumulation and activation, is a major pathogenesis of NP. Microglia are the resident immune cells in central nervous system and play as stimulative promoter in various diseases. The activated microglia at the early stages may be e beneficial to the host during the neurodegenerative lesion, yet longstanding microglial overactivation would induce chronic inflammation leading to pathological pain by releasing several cytokines, enzymes, and mediators. Besides, uncontrolled microglial overactivation could cause secondary neuronal damage and even death by these substances; therefore, increasing researchers have devoted themselves to the investigation concerning anti-inflammatory targets because might help discover a novel treatment of NP. Recently, a study suggested that LDL receptor-related protein-1 deficiency in microglia could block neuroinflammation and attenuate NP processing [27]. For the past few years, Src has been reported to be closely related to searching treatment for blocking neuroinflammation [28,29]. Socodato and his workmates found that Src played a key role in triggering microglial activation [20]. More notable thing is that modulating Src/ERK signaling alleviates the generation of PGE_2_ and NO in LPS-induced BV2 microglia [29]. Additionally, Src/ERK signaling provoked inspiratory resistive breathing induced acute lung injury and inflammation [30]. Yang et al. [28] suggested that regulating Src signaling pathway may reduce microglia-induced neuroinflammation in Parkinson’s disease. All above studies showed Src is a crucial factor involving in neuroinflammatory development. In our study, we found that Src expression was significantly increased in LPS-induced BV2 microglia and spinal cords of PNL-treated mice; besides, we demonstrated that p-Src after PP2 treatment was significantly reduced in both *in vitro* and *in vivo* studies, which was consistent with the results in mouse brain treated with PP2 in a previous study [28]. *In vitro*, LPS-induced microglia treated with PP2 showed lower activation and infiltration ability accompanied by alleviated neuroinflammation. Mechanically, NF-κB pathway, as a canonical pro-inflammatory pathway, was proved to be inhibited the nuclear translocation in LPS-induced microglia after PP2 treatment; thus, the iNOS and COX-2 expression as well as pro-inflammatory cytokines, such as TNF-α and IL-6, were reduced in the activated microglia that were treated with PP2. Consistently, we witnessed a significant decrease in the above pro-inflammatory mediators in spinal cords of PNL-treated mice. Moreover, the microglial activation and accumulation were inhibited by the treatment of Src inhibition. Although previous studies have been noticed the elevated expression of Src in NP processing and the decrease of Src expression after anti-inflammatory treatment in NP model [31–33], they hardly explained the results and clarified the underlying mechanism in NP. In our article, we specifically utilized the Src inhibitor PP2 to treat LPS-induced microglia and PNL-treated mice, and systematacially indicated the effect of Src in neuroinflammation and NP. More importantly, we proved that the inhibition of Src in microglia and NP mouse model could reduce the degree of neuroinflammation and prevent the pian-induced functional impairment after PNL treatment. Therefore, our findings support that Src may be a potential target in therapeutic strategy against neuroinflammation-induced NP.

## Conclusions

The current study demonstrated that Src inhibition reduced LPS-induced microglial neuroinflammation *via* inhibiting NF-κB nuclear translocation. Moreover, Src inhibition attenuated microglial activation and accumulation during neuroinflammation in PNL-induced NP model.

## Highlights

1. Src inhibitor reduces BV2 microglial activation by LPS stimulation
2. Src inhibition inhibits microglia-driven inflammation via blocking NF-κB activity
3. Src inhibition reduces neuroinflammation and improves allodynia after PNL
